# Gender differences in the relationships between dietary phytosterols intake and prevalence of obesity in Chinese population

**DOI:** 10.1002/fsn3.3097

**Published:** 2022-10-13

**Authors:** Panpan Guo, Rennan Feng, Zixiang Li, Ting Han

**Affiliations:** ^1^ Department of Clinical Nutrition Shanghai Tenth People's Hospital, Tongji University School of Medicine Shanghai China; ^2^ Shanghai Clinical Nutrition Quality Control Center Shanghai China; ^3^ Department of Nutrition and Food Hygiene, Public Health College Harbin Medical University Harbin China

**Keywords:** dietary phytosterols, human, internet‐based dietary questionnaire for Chinese, obesity

## Abstract

To investigate the associations between different phytosterols (PSs) intake and subtype of obesity in Chinese. Total 6073 adults aged ≥18 years was enrolled from China. General characteristics were completed by the validated dietary questionnaire. For total phytosterols intake, comparing Q4 with Q1 was inversely associated with the risks of overweight [odds ratio (OR) 95% confidence interval (CI), 0.82 (0.69, 0.96), *p* < .05]. The intake of stigmasterol, β‐sitosterol, β‐sitostanol and campestanol were associated with the lower risks of obesity, whereas no significant correlationss were found between campesterol intake and any subtype of obesity in the multivariable‐adjusted model. Interestingly, the stigmasterol intake was inversely related with the prevalence of central obesity in female, while the β‐sitostanol intake was found in male [OR 95% CI in Q3 of 0.78 (0.60–0.99) and 0.71 (0.56–0.91), respectively; *p* < .05]. The multiple linear regression models showed that fruits, vegetable‐oil, nuts and seeds may be important diet sources of PSs. The intake of total PSs, β‐sitosterol, stigmasterol, β‐sitostanol and campestanol were inversely associated with the prevalence of obesity. Moreover, the lower obesity risk for total PSs and PSs subgroups differed for the gender. The firm results deserve to be further verified in cohort studies.

## Graphical abstract

The present study suggests that the intake of total phytosterols (PSs), stigmasterol, ß‐sitosterol, ß‐sitostanol and campestanol were inversely associated with the prevalence of obesity. In addition, lower obesity risk for total PSs and PSs subgroups differed for the gender. These results deserve to be further verified in cohort studies.

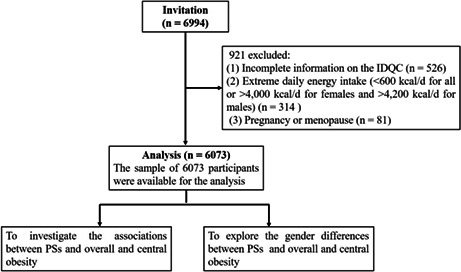

## 1 INTRODUCTION

Obesity has become an increasing challenge to public health, and China has the largest number of affected people worldwide, with about 16.4% and 34.3% of Chinese adults being obese and overweight, respectively (Zeng et al., 2021). Overweight and obesity were the sixth leading risk factor for disability and death combined in Global Burden of Disease Study 2019 (GBD 2019 Risk Factors Collaborators, 2020). The annual cost of overweight and obesity in China was as high as 24.35 billion Yuan, accounting for 2.46% of the total health care cost (Qin & Pan, 2016). Overweight and obesity are linked to increased risks of non‐communicable diseases (NCDs) including diabetes, hypertension, cardiovascular diseases (CVDs) and certain cancers (Li et al., 2018; Zeng et al., 2021). Dietary habits were considered to have a significant impact on development and prevention of NCDs (Han et al., 2021). Therefore, it is necessary to pay attention to the associations between daily diet and NCDs, and raise the healthy and reasonable dietary recommendation.

The phytosterols (PSs, including plant sterols and plant stanols) are the essential non‐nutritive but bioactive components in plant‐based foods, the structures of which are 28‐ or 29‐carbon alcohols with similar to cholesterol (Ghaedi et al., 2019). According to different food sources, it is mainly divided into β‐sitosterol, stigmasterol and campesterol, with β‐sitosterol as the dominant component, accounting for 60%–90% of the total PSs content (Guo & Han, 2021). Depending on the saturation state of the C‐5 bond, PSs are divided into sterols and stanols. The β‐sitostanol and campestanol are structures with saturated double bond of β‐sitosterol and campesterol, respectively. Some studies reported that PS mixture intake has no effect on the susceptibility to obesity in mice model (Feng et al., 2020). However, the majority of evidences have suggested that PSs supplementation can significantly decrease triglyceride (TG), total cholesterol (TC) levels, fat absorption, and increase fecal lipid excretion as well thereby reducing body weight (Ghaedi et al., 2019). Recently, the meta‐analysis of randomized controlled trials revealed that body mass index (BMI) significantly decreased following PSs supplementation (Ghaedi et al., 2019). Other possible anti‐obesity effects of PSs could be by improving insulin sensitivity, increasing adiponectin levels and by modulating AMP‐activated protein kinase (AMPK) activation and phospholipase A2 inhibition (Ghaedi et al., 2019; Xiong et al., 2018). The clinical trials found that 4 weeks PSs supplementation (1.6 g/day) could prevent obesity‐associated inflammation by suppressing the expression of the C‐C motif chemokine 2, monocyte chemotactic protein‐1 (MCP‐1), and interleukin‐10‐R and (Lambert et al., 2017) and decreasing circulating IL‐6 and TNF‐α (Kurano et al., 2018). Particularly, β‐sitosterol isolated from Moringa oleifera reduced the secretion of IL‐6, IL‐1β, IL‐8 and TNF‐αin LPS‐stimulated human keratinocytes and mouse macrophages (Liao et al., 2018). Another study reported the stigmasterol significantly reduced body weight comparing with mice fed high‐fat diet at 17 weeks. However, 𝛽‐sitosterol was not as effective as stigmasterol (Feng, Dai, et al., 2018). Stigmasterol decreases the intestinal bile acid levels that in turn decrease lipid absorption from diet, thereby attenuating weight gain (Feng, Gan, et al., 2018). Body weight, adipose expansion, and fatty liver changes are significantly suppressed with markedly elevated circulating sitosterol levels in mice (Kurano et al., 2018). In conclusion, although some studies have reported that PS mixture intake has no effect on obesity, some individual PS such as β‐sitosterol, stigmasterol and sitosterol has impact on preventing obesity and related diseases. Studies on anti‐obesity properties of PSs are controversial. Further researches are warranted to clarify whether individual PS may be protective against obesity.

Our previous study conducted in Chinese population have shown beneficial effect of PSs intake on obesity (Li et al., 2018). However, limited evidence is available for large Chinese populations regarding the relationships between PSs intake and obesity. Moreover, there is few evidence revealed some details such as PSs type and differences between genders were not considered in the effects of PSs intake on obesity. Therefore, the aim of this study to examine the associations between the intake of different types of PSs and the prevalence of obesity in northern Chinese population. We focused on the five most commonly PSs types (stigmasterol, β‐sitosterol, campesterol, β‐sitostanol and campestanol) in order to investigate the associations and gender differences between these and overall and central obesity. In addition, gender difference was also explored.

## 2 METHODS

### 2.1 Study population

We used data from the validated internet‐based dietary questionnaire for Chinese (IDQC, www.yyjy365.org/diet; Feng et al., 2016). This cross‐sectional study included 6994 adults aged ≥18 years, were enrolled for this study from March 2014 to December 2016 in northern Chinese area. The sample size (*n*) was calculated as *n* = *t*_α_^2^ × *p* × *q*/*e* ^2^, where *p* is the percentage of the study characteristic, *q* is calculated as 1 − *p*, *e* is the accepted margin of error (set at 0.05), and *t*_α_ = 1.96 (95% CI) (Rodriguez Del Aguila & Gonzalez‐Ramirez, 2014). The exclusive criteria were as follows: (1) incomplete information on the IDQC (*n* = 526); (2) extreme daily energy intake (<600 kcal/day for all or >4000 kcal/day for females and >4200 kcal/day for males) (*n* = 314); (3) pregnancy or menopause (*n* = 81). Finally, the sample of 6073 participants were available for the analysis.

This study was conducted with the approval by Ethics Committee of Harbin Medical University and signed informed consents from all participants, and in accordance with the ethical standards in the Declaration of Helsinki.

### 2.2 Estimation of dietary phytosterols intake

Dietary exposure was measured by the validated IDQC. All participants were invited to complete the IDQC for the past 4 months. The clickable images were created as references to aid participants to estimate the food portions. Participants could click the checkbox to choose the suitable food and the frequency and amount of food intake in the IDQC. According to the dietary habits of Chinese residents, commonly foods were divided into 16 categories (i.e., grains, legumes, potatoes, vegetables, fruits, seeds and nuts, fungus, livestock, poultry, eggs, fish, dairy, snacks, condiments, sugar and beverages) in the Chinese food composition table were chosen for measurement. The contents of stigmasterol, β‐sitosterol, campesterol, β‐sitostanol and campestanol in food were analyzed at Institute of Nutrition and Food Safety, Chinese Centre for Disease Control and Prevention, Beijing, China, using gas chromatograph methods by Han et al. (2007, 2009). The database of PSs was established and then estimated PSs intake using the IDQC.

### 2.3 Anthropometric measurements, the definition of overweight, obesity and central obesity

Bodyweight, height, and waist circumference (WC) of participants were measured by well‐trained nurses. Participants were asked to wear light, thin clothing and no shoes. Bodyweight, height and WC were measured to the nearest 0.1 kg, 0.1 cm and 0.1 cm, respectively. Body mass index (BMI) in kg/m^2^ was calculated as body weight (kg) in kilograms divided by the square of the height (m). According to the 2006 Guidelines on Preservation and Control Overweight and Obesity in Chinese Adults classification (Li et al., 2018), Overweight, obesity and central obesity were defined as 24 ≤ BMI < 28 kg/m^2^, BMI ≥ 28 kg/m^2^, WC ≥ 80 cm (for females) and WC ≥ 85 cm (for males), respectively.

### 2.4 Other covariates

Other covariates involved age, gender, education, incomes, physical exercise and smoking status. These data were also gained by the IDQC. For instance, education was divided into college and postgraduate and above, senior high school, junior high school, primary school and below. Income was divided into <2000 Yuan, 2000–4000 Yuan, >4000 Yuan. Smoking was divided into >20 times/day, 10–20 times/day, 1–9 times/day, < once/day, quit smoking and never smoking. Quit smoking was defined abstinent for at least 30 days before the enrollment to the study.

### 2.5 Statistical analysis

All continuous variables and categorical variables are presented as means ± SD and the percentage, separately. Chi‐square test was used to compare the differences in frequency of categorical variables. One‐way analysis of variance (one‐way ANOVA) or Kruskal‐Wallis test was used to compare the means of continuous variables. Multivariable logistic regression analyses were used to exploring relationships between dietary phytosterols intake and different types of obesity. Age, gender, education, incomes, physical exercise and smoking status were adjusted in models. All analyses were carried out with SPSS Statistics ver. 22.0 (IBM). The level of significance was set at *p* < .05.

## 3 RESULTS

### 3.1 Participant characteristics and dietary PSs intake according to quartiles of total PSs intake

The characteristics and dietary PSs intake of participants were summarized in Table 1. Participants were classified into quartiles of total PSs intake. The lowest quartile of consumption (Q1) was considered as the reference category. The phytosterols intake was higher in females than male. Participants of Q4 tended to have higher levels of physical activity, education and income, but lower levels of alcohol intake and smoking versus the first quartile. However, participants in Q4 were less likely to have obesity and central obesity (*p* < .05 for all).

**TABLE 1:** General characteristics of participants according to quartiles of total PSs intake

	Q1	Q2	Q3	Q4	*p*‐value^a^
	<715 mg/day	715–1175 mg/day	1175–1787 mg/day	>1787 mg/day	
Total, *n*	1519	1518	1518	1518	
Total phytosterols, mg/day	491.90 (357.96, 611.56)	923.38 (809.24, 1040.02)	1472.67 (1311.23, 1617.09)	2474.53 (2059.85, 3171.54)	<.001
Stigmasterol, mg/day	35.25 (24.24, 47.39)	56.58 (44.87, 71.31)	77.39 (63.46, 97.66)	120.42 (92.39, 155.35)	<.001
β‐sitosterol, mg/day	276.90 (196.11, 345.40)	539.37 (473.76, 618.67)	902.72 (785.30, 1039.04)	1538.12 (1293.13, 2023.00)	<.001
Campesterol, mg/day	30.833 (22.59, 40.26)	56.83 (44.96, 68.59)	90.08 (71.14, 106.10)	141.76 (116.17, 195.95)	<.001
β‐sitostanol, mg/day	21.97 (13.73, 31.36)	47.82 (33.99, 64.08)	88.78 (62.42, 124.90)	159.92 (125.43, 228.04)	<.001
Campestanol, mg/day	82.45 (47.71, 126.34)	167.26 (115.32, 236.60)	224.62 (140.61, 337.68)	383.27 (249.40, 585.40)	<.001
Male (*n* %)	639 (42.07%)	585 (38.54%)	592 (39.00%)	559 (36.82%)	<.05
Age, years	38.47 ± 16.28	40.42 ± 15.74	40.89 ± 15.70	38.70 ± 14.90	.033
Energy, kcal	1829.18 ± 741.08	2132.58 ± 701.83	2375.92 ± 710.93	2872.02 ± 687.43	<.001
BMI, kg/m^2^	22.57 ± 3.31	22.93 ± 3.28	23.06 ± 3.51	22.92 ± 3.25	.160
WC, cm	78.21 ± 9.88	78.62 ± 9.73	78.65 ± 9.36	78.15 ± 9.53	.203
Overweight/obese, *n* (%)	475 (31.27%)	529 (34.85%)	537 (35.38%)	523 (34.45%)	.074
Overweight, *n* (%)	384 (25.28%)	416 (27.40%)	404 (26.61%)	427 (28.12%)	.327
Obesity, *n* (%)	92 (6.06%)	115 (7.58%)	135 (8.89%)	99 (6.52%)	.013
Central obesity, *n* (%)	480 (31.60%)	528 (34.78%)	553 (36.43%)	519 (34.19%)	.043
Education level, *n* (%)					
Primary school and below	97 (6.39%)	133 (8.76%)	73 (4.81%)	45 (2.96%)	<.001
Junior high school	193 (12.71%)	236 (15.55%)	231 (15.22%)	160 (10.54%)	
Senior high school	265 (17.45%)	254 (16.73%)	333 (21.94%)	334 (22.00%)	
College	918 (60.43%)	850 (55.99%)	847 (55.80%)	928 (61.13%)	
Postgraduate and above	46 (3.03%)	45 (2.96%)	34 (2.24%)	51 (3.36%)	
Smoking status, *n* (%)					
Never smoked	1260 (82.95%)	1296 (85.38%)	1318 (86.82%)	1335 (87.94%)	.002
Former smoker	69 (4.54%)	56 (3.69%)	44 (2.90%)	56 (3.69%)	
Current smoker	190 (12.51%)	166 (10.94%)	156 (10.28%)	127 (8.37%)	
Alcohol consumption, *n* (%)	248 (16.33%)	247 (16.27%)	227 (14.95%)	181 (11.92%)	.002
Physically active, *n* (%)	740 (48.72%)	764 (50.33%)	848 (55.86%)	891 (58.70%)	<.001
Income per month, Yuan					
<2000	547 (36.01%)	473 (31.16%)	378 (24.90%)	394 (25.96%)	<.001
2000–4000	808 (53.19%)	860 (56.65%)	939 (61.86%)	909 (59.88%)	
>4000	164 (10.80%)	185 (12.19%)	201 (13.24%)	215 (14.16%)	

*Note*: Data are presented as the mean ± SD, median [interquartile range], or as *n* (%).

Abbreviations: BMI, body mass index; WC, waist circumference.

^a^ *p*‐values were determined using χ^2^ tests for categorical variables, analysis of covariance (ANCOVA) for continuous variables, and the Kruskal‐Wallis test for continuous variables that were not normally distributed.

### 3.2 Odds ratios (OR) and 95% confidence intervals (CI) for different types of obesity according to total PSs intake

As shown in Table 2, with adjustment for age, sex, education, income, physical activity, smoking status and alcohol intake, for total phytosterols intake, comparing Q2 with Q1 was linked with lower prevalence of overweight/obesity, overweight and central obesity [OR 95% CI in Q2 of 0.81 (0.69–0.96), 0.84 (0.71–0.99) and 0.83 (0.69–0.97), respectively; all *p* < .05]. For stigmasterol, we found higher dietary stigmasterol intake was linked with lower prevalence of overweight/obesity, overweight and central obesity. For β‐sitosterol, participants in Q2 and Q4 were less likely to have overweight/obesity and overweight, respectively. Participants in Q3 of β‐sitostanol tended to have lower prevalence for central obesity [OR 95% CI, 0.79 (0.67–0.94)] but campestanol for overweight/obesity and overweight [OR 95% CI, 0.78 (0.66–0.93); 0.77 (0.64–0.91), respectively]. However, no significant associations were found between campesterol intake and any subtype of obesity in the multivariable‐adjusted model.

**TABLE 2:** Odds ratios (ORs) and 95% confidence intervals (CIs) of obesity according to quartiles (Q1–Q4) of different PSs intake

	Quartiles based on different phytosterols intake			
	Q1	Q2	Q3	Q4
Total phytosterols				
Overweight/obesity	1	0.81 (0.69, 0.96)*	0.89 (0.75, 1.04)	0.91 (0.77.1.07)
Overweight	1	0.84 (0.71, 0.99)*	0.86 (0.73, 1.02)	0.82 (0.69, 0.96)*
Obesity	1	0.87 (0.64, 1.18)	1.06 (0.79, 1.41)	1.28 (0.97, 1.68)
Central obesity	1	0.83 (0.69, 0.97)*	0.87 (0.74, 1.03)	0.95 (0.80, 1.12)
Stigmasterol				
Overweight/obesity	1	0.82 (0.70, 0.97)*	0.79 (0.67, 0.93)*	0.84 (0.71, 0.98)*
Overweight	1	0.81 (0.69, 0.97)*	0.79 (0.67, 0.94)*	0.87 (0.74, 1.03)
Obesity	1	0.98 (0.74, 1.31)	1.01 (0.78, 1.33)	0.93 (0.70, 1.23)
Central obesity	1	0.81 (0.69, 0.96)*	0.82 (0.69, 0.97)*	0.80 (0.67, 0.94)*
β‐sitosterol				
Overweight/obesity	1	0.83 (0.70, 0.98)*	0.93 (0.79, 1.05)	0.91 (0.77, 1.07)
Overweight	1	0.88 (0.74, 1.05)	0.88 (0.75, 1.05)	0.84 (0.71, 0.99)*
Obesity	1	0.77 (0.57, 1.04)	1.13 (0.86, 1.49)	1.19 (0.91, 1.57)
Central obesity	1	0.85 (0.72, 1.01)	0.89 (0.75, 1.05)	0.99 (0.84, 1.17)
Campesterol				
Overweight/obesity	1	0.88 (0.75, 1.04)	0.95 (0.81, 1.12)	1.00 (0.85, 1.18)
Overweight	1	0.93 (0.78, 1.10)	0.90 (0.76, 1.07)	0.96 (0.81, 1.13)
Obesity	1	0.87 (0.65, 1.16)	1.10 (0.83, 1.46)	1.11 (0.84, 1.46)
Central obesity	1	0.88 (0.74, 1.04)	0.92 (0.77, 1.08)	0.99 (0.84, 1.170)
β‐sitostanol				
Overweight/obesity	1	0.94 (0.80, 1.11)	0.94 (0.79, 1.10)	0.91 (0.77, 1.03)
Overweight	1	1.01 (0.85, 1.20)	0.91 (0.77, 1.08)	0.91 (0.77, 1.07)
Obesity	1	0.79 (0.59, 1.06)	1.04 (0.78, 1.36)	0.97 (0.74, 1.28)
Central obesity	1	0.92 (0.78, 1.09)	0.79 (0.67, 0.94)*	1.01 (0.86, 1.19)
Campestanol				
Overweight/obesity	1	0.88 (0.74, 1.04)	0.78 (0.66, 0.93)*	1.04 (0.88, 0.93)
Overweight	1	0.89 (0.75, 1.06)	0.77 (0.64, 0.91)*	1.03 (0.87, 1.22)
Obesity	1	0.91 (0.68, 1.23)	1.01 (0.76, 1.35)	1.10 (0.83, 1.46)
Central obesity	1	0.97 (0.81, 1.15)	0.97 (0.82, 1.15)	1.08 (0.91, 1.27)

*Note*: Multivariate model adjusted for age, sex, education, income, physical activity, smoking status, and alcohol intake.

* *p* < .05 compared with the 1st quartile.

### 3.3 Multivariate adjusted OR and 95% CI of different types of obesity by category of PSs stratified by gender

Tables 3 and 4 provide the correlations between dietary PSs intake and prevalence of obesity for female and male, respectively. In female, participants consuming the higher levels of campestanol (Q3 and Q4) revealed significant inverse associations in overweight/obesity and overweight. In comparison with the 1st quartile, the multivariable‐adjusted ORs of central obesity of the 3th quartile were 0.78 (0.61, 0.99) and of overweight were 0.72 (0.57, 0.91) for stigmasterol (All *p* < .05). However, compared with the 1st quartile, the multivariable‐adjusted ORs of central obesity of the 3th quartile were 0.71 (0.56, 0.91) for β‐sitostanol and of overweight 0.75 (0.58, 0.96) for campestanol in male (All *p* < .05).

**TABLE 3:** Odds ratios (ORs) and 95% confidence intervals (CIs) of obesity according to quartiles (Q1–Q4) of different PSs intake in female

	Quartiles based on different phytosterols intake (*n* = 3698)			
	Q1	Q2	Q3	Q4
Total phytosterols				
Overweight/obesity	1	0.90 (0.72, 1.14)	0.96 (0.68, 1.09)	0.94 (0.75, 1.18)
Overweight	1	0.87 (0.69, 1.11)	0.85 (0.67, 1.07)	0.83 (0.66, 1.05)
Obesity	1	1.02 (0.65, 1.59)	1.02 (0.66, 1.58)	1.44 (0.96, 2.17)
Central obesity	1	0.81 (0.63, 1.04)	0.83 (0.64, 1.05)	1.02 (0.80, 1.29)
Stigmasterol				
Overweight/obesity	1	0.79 (0.63, 1.01)	0.73 (0.58, 0.92)*	0.85 (0.68, 1.07)
Overweight	1	0.80 (0.63, 1.01)	0.72 (0.57, 0.91)*	0.86 (0.69, 1.09)
Obesity	1	0.90 (0.58, 1.39)	1.05 (0.70, 1.57)	0.98 (0.65, 1.47)
Central obesity	1	0.77 (0.60, 0.99)*	0.78 (0.61, 0.99)*	0.91 (0.72, 1.16)
β‐sitosterol				
Overweight/obesity	1	0.82 (0.65, 1.04)	0.96 (0.77, 1.21)	0.82 (0.66, 1.03)
Overweight	1	0.86 (0.68, 1.09)	0.95 (0.75, 1.19)	0.79 (0.63, 1.00)
Obesity	1	0.75 (0.48, 1.17)	1.01 (0.67, 1.52)	1.09 (0.73, 1.63)
Central obesity	1	0.83 (0.65, 1.056)	0.87 (0.69, 1.11)	1.01 (0.80, 1.28)
Campesterol				
Overweight/obesity	1	0.89 (0.71, 1.12)	0.93 (0.74, 1.17)	0.91 (0.72, 1.14)
Overweight	1	0.89 (0.70, 1.12)	0.87 (0.69, 1.10)	0.87 (0.69, 1.09)
Obesity	1	0.89 (0.58, 1.38)	1.19 (0.79, 1.82)	1.17 (0.78, 1.77)
Central obesity	1	0.83 (0.65, 1.06)	0.89 (0.70, 1.14)	1.03 (0.82, 1.31)
β‐sitostanol				
Overweight/obesity	1	0.96 (0.76, 1.22)	0.94 (0.75, 1.18)	0.88 (0.71, 1.10)
Overweight	1	0.99 (0.79, 1.26)	1.03 (0.81, 1.29)	0.94 (0.74, 1.18)
Obesity	1	0.81 (0.53, 1.22)	0.79 (0.53, 1.19)	0.82 (0.55, 1.22)
Central obesity	1	0.87 (0.68, 1.11)	0.88 (0.69, 1.12)	1.06 (0.84, 1.33)
Campestanol				
Overweight/obesity	1	0.88 (0.69, 1.11)	0.71 (0.56, 0.89)*	0.79 (0.63, 0.99)*
Overweight	1	0.87 (0.69, 1.10)	0.73 (0.57, 0.92)*	0.80 (0.61, 0.98)*
Obesity	1	0.93 (0.60, 1.44)	0.93 (0.60, 1.43)	1.04 (0.68, 1.60)
Central obesity	1	0.95 (0.74, 1.21)	0.89 (0.69, 1.14)	0.99 (0.77, 1.26)

*Note*: Multivariate model adjusted for age, education, income, physical activity, smoking status, and alcohol intake.

* *p* < .05 compared with the 1st quartile.

**TABLE 4:** Odds ratios (ORs) and 95% confidence intervals (CIs) of obesity according to quartiles (Q1–Q4) of different PSs intake in male

	Quartiles based on different phytosterols intake (*n* = 2375)			
	Q1	Q2	Q3	Q4
Total phytosterols				
Overweight/obesity	1	0.82 (0.64, 1.04)	0.93 (0.73, 1.18)	0.98 (0.78, 1.25)
Overweight	1	0.86 (0.66, 1.10)	0.90 (0.70, 1.16)	0.91 (0.71, 1.17)
Obesity	1	0.83 (0.55, 1.25)	1.05 (0.71, 1.54)	1.16 (0.80, 1.68)
Central obesity	1	0.83 (0.65, 1.06)	0.89 (0.70, 1.13)	0.86 (0.68, 1.10)
Stigmasterol				
Overweight/obesity	1	0.91 (0.71, 1.15)	0.92 (0.72, 1.16)	0.92 (0.73, 1.17)
Overweight	1	0.86 (0.67, 1.11)	0.91 (0.71, 1.16)	0.89 (0.69, 1.13)
Obesity	1	1.15 (0.78, 1.70)	1.03 (0.69, 1.52)	1.16 (0.79, 1.71)
Central obesity	1	0.89 (0.70, 1.14)	0.84 (0.66, 1.07)	0.79 (0.63, 1.01)
β‐sitosterol				
Overweight/obesity	1	0.86 (0.67, 1.09)	0.92 (0.72, 1.16)	0.98 (0.78, 1.25)
Overweight	1	0.91 (0.71, 1.17)	0.79 (0.61, 1.01)	0.87 (0.68, 1.11)
Obesity	1	0.79 (0.51, 1.23)	1.42 (0.97, 2.09)	1.36 (0.93, 1.99)
Central obesity	1	0.85 (0.66, 1.08)	0.95 (0.75, 1.21)	0.93 (0.73, 1.18)
Campesterol				
Overweight/obesity	1	0.97 (0.76, 1.23)	0.97 (0.76, 1.23)	1.11 (0.87, 1.41)
Overweight	1	0.98 (0.76, 1.26)	0.94 (0.73, 1.21)	1.05 (0.82, 1.35)
Obesity	1	0.92 (0.62, 1.38)	1.05 (0.71, 1.55)	1.13 (0.77, 1.65)
Central obesity	1	0.98 (0.77, 1.24)	0.96 (0.75, 1.22)	0.96 (0.75, 1.22)
β‐sitostanol				
Overweight/obesity	1	0.97 (0.76, 1.23)	0.87 (0.68, 1.11)	0.92 (0.73, 1.17)
Overweight	1	1.05 (0.82, 1.35)	0.78 (0.60, 1.00)	0.89 (0.69, 1.14)
Obesity	1	0.81 (0.54, 1.23)	1.26 (0.86, 1.84)	1.13 (0.77, 1.66)
Central obesity	1	0.99 (0.78, 1.27)	0.71 (0.56, 0.91)*	0.97 (0.76, 1.23)
Campestanol				
Overweight/obesity	1	0.76 (0.60, 0.97)*	0.73 (0.57, 0.93)*	0.99 (0.78, 1.26)
Overweight	1	0.79 (0.62, 1.02)	0.75 (0.58, 0.96)*	1.00 (0.78, 1.28)
Obesity	1	0.83 (0.56, 1.22)	0.84 (0.57, 1.23)	0.94 (0.65, 1.37)
Central obesity	1	0.89 (0.70, 1.14)	0.95 (0.75, 1.21)	0.96 (0.76, 1.22)

*Note*: Multivariate model adjusted for age, education, income, physical activity, smoking status, and alcohol intake.

* *p* < .05 compared with the 1st quartile.

### 3.4 The multiple linear regression for phytosterol‐related food analysis

Table 5 presents the multiple linear regression for phytosterol‐related food analysis. The multiple linear regression reported there are closely linear correlations between fruits, vegetable‐oil, nuts and seeds and plant sterols (*R* = .91, *F* = 4151.28, *p* < .001).

**TABLE 5:** The multiple linear regression in PSs

Model	Unstandardized coefficients		Standardized coefficients	*t*	*p*‐value
	*B*	SE	*β*		
(Constant)	11.931	15.769		0.757	.449
Grains	0.109	0.036	.017	3.054	.002
Potatoes	0.296	0.111	.016	2.676	.007
Soy food	0.267	0.074	.021	3.598	<.001
Vegetables	0.439	0.028	.103	15.662	<.001
Fruits	1.779	0.026	.404	68.812	<.001
Nuts and seeds	15.544	0.140	.647	111.289	<.001
Vegetable‐oil	1.004	0.416	.013	2.411	.016

*Note*: *R* = .91, *F* = 4151.28, *p* < .001.

## 4 DISCUSSION

We applied an internet‐based dietary questionnaire to in a large northern Chinese population to investigate the associations between PSs intake and the prevalence of obesity incidence. Our findings indicated: (1) The intake of total PSs, β‐sitosterol, stigmasterol, β‐sitostanol, campestanol were associated with lower risks of obesity, whereas no significant associations were observed between campesterol intake and any subtype of obesity; Besides, the reverse relationships were observed between dietary PSs and central obesity. (2) total PSs and subgroups PSs intake were different for female and male in prevention of obesity risks. The higher levels of campestanol were associated with the lower risk of overweight/obesity and overweight in female and male. Interestingly, the stigmasterol intake was inversely related with the prevalence of central obesity in female, while the β‐sitostanol intake was found in male. (3) The fruits, vegetable‐oil, nuts and seeds may be important diet sources of PSs.

In line with our previous findings, PSs intake was associated with the lower risk of obesity. Similarly, the recent meta‐analysis including 79 randomized controlled trials suggested PSs intake in subjects with BMI ≥25 and hyperlipidemic significantly decreased body weight and BMI (Han et al., 2021). Also, the majority of evidences revealed β‐sitosterol, one of the most abundant PSs, could inhibit obesity by the downregulation of c‐Jun‐N‐terminal kinase (JNK) and IKKβ/NF‐κB signaling pathway, thereby reducing the adipose tissue mass and inhibiting the preadipocytes proliferation (Jayaraman et al., 2021; Vezza et al., 2020). Moreover, β‐sitosterol could compete with cholesterol at a receptor cholesterol‐binding site and shift its conformation toward normal, which may affect the type 1 cholecystokinin receptor (CCK1R) function (Desai et al., 2016). Further studies are warranted to investigate the possible beneficial effects of β‐sitosterol in obesity. For stigmasterol, stigmasterol supplementation suppressed cholesterol synthesis and hepatic bile acid through stimulating transintestinal cholesterol excretion independent of liver X receptor activation in the small intestine in rats (Lifsey et al., 2020). Interestingly, the animal experiments have reported that with β‐sitosterol and stigmasterol supplementation could ameliorate the intestinal dysbiosis, thereby increasing cholesterol excretion, together with decreasing in esterified cholesterol levels and serum non‐HDL cholesterol (Vezza et al., 2020). However, no significant relationships were found between campesterol intake and obesity in the present study. Few data are available for the relationships between campesterol intake and obesity. Gaps in the evidence base a suggest that more research is urgently needed, especially relating to the associations between the intake of specific types of PSs and subtype of obesity.

There is a gender difference in the prevalence of obesity. The global incidence of obesity is higher in female than in male in 141 countries in 2014 (Mauvais‐Jarvis, 2017; NCD Risk Factor Collaboration (NCD‐RisC), 2016). Interestingly, in recent decades, the prevalence of central obesity has increased more in female than in male in the USA (Mauvais‐Jarvis, 2017). Therefore, the Multivariable logistic regression analyses were conducted to further investigate the gender differences of the relationships between PSs intake and the different subtypes of obesity. In gender‐stratified analysis, the higher levels of campestanol were linked with the lower risk of overweight/obesity and overweight in female and male. However, the stigmasterol intake was associated with the prevalence of central obesity in female, while the β‐sitostanol intake was found in male. The recent majority of studies suggested that the prevalence of obesity might have influenced the positive effect of PSs intake on obesity. However, scarce data have investigated above associations differed between female and male. Further studies are warranted to confirm these observational findings and explain the observed gender‐specific differences.

The most important natural PSs dietary sources are nuts, vegetable oils, grains, and grain‐derived products in human diet (Feng et al., 2020). Consistently, In the present study, our study showed the associations between fruits, vegetable‐oil, nuts and seeds and plant sterols (*R* = .91, *F* = 4151.28, *p* < .001). The average daily intakes of total PSs usually range from 200 to 400 mg in general population and high daily intake of 500–1000 mg can be obtained by vegetarians (Ras & Trautwein, 2017). However, the bioavailability of PSs is very low, which only about 2%–6% can be absorbed through intestine and the left will be excluded into the feces. Most RCT studies give evidence that the health benefits are obtained at the high intake of PSs (around 2 g/day) (Feng et al., 2020), which cannot be achieved in habitual diets. However, in most RCTs, the health benefits are observed at a high dose of phytosterols intake (about 2 g/day), which cannot be achieved in habitual diets. Further research is worth to investigate the safety and bioavailability issues of PSs and the derivatives in clinical trials.

To our knowledge, this study is the first to investigate the associations between different PSs intake and subtype of obesity. Most importantly, we found total PSs and subgroups PSs intake were different for female and male in prevention of obesity risks. Furthermore, the present study used multiple linear regression modeling for the dietary‐related factors to investigate the potential mechanisms linking PSs intake and obesity. The results of the study should be interpreted with some limitations. First, the incidence of obesity was ascertained on the basis of self‐reported information, therefore, information biases could be existed. In spite of recall bias is another limitation, clickable images were conducted for each food as a reference to assist in the estimation of food portions. Moreover, the cross‐sectional nature of the data precludes conclusions about causality or its direction. Therefore, the results should be further drawn in further investigation.

## 5 CONCLUSION

In conclusion, the present study suggests that the intake of total PSs, stigmasterol, β‐sitosterol, β‐sitostanol and campestanol were inversely associated with the prevalence of obesity. In addition, lower obesity risk for total PSs and PSs subgroups differed for the gender. These results deserve to be further verified in cohort studies.

## FUNDING INFORMATION

Funding was supported by the National Natural Science Hospital Cultivation Fund of China (No. 04.03.19.153).

## CONFLICT OF INTEREST

None.

## ETHICAL APPROVAL

This study was approved by the Human Research Ethics Committee of the Harbin Medical University ([2015] 006).

## INFORMED CONSENT

Written informed consent was obtained from all participants.

## Data Availability

Data are available upon request from corresponding author.
